# Lack of Evidence That Bird Feeders Are a Source of Salmonellosis during Winter in Poland

**DOI:** 10.3390/ani11061831

**Published:** 2021-06-19

**Authors:** Martyna Frątczak, Piotr Indykiewicz, Beata Dulisz, Jacek J. Nowakowski, Tomasz Janiszewski, Jan Szeptycki, Jarosław Wilczyński, Piotr Tryjanowski

**Affiliations:** 1 Department of Zoology, Poznań University of Life Sciences, Wojska Polskiego 71C, PL-60-625 Poznań, Poland; martynafrt@gmail.com; 2 Department of Biology and Animal Environment, University of Technology and Life Sciences, Mazowiecka 28, PL-85-084 Bydgoszcz, Poland; inpio@poczta.onet.pl; 3 Department of Ecology & Environmental Protection, University of Warmia and Mazury in Olsztyn, Plac Łódzki 3, PL-10-727 Olsztyn, Poland; beata.dulisz@uwm.edu.pl (B.D.); jacek.nowakowski@uwm.edu.pl (J.J.N.); 4 Department of Teacher Training and Biodiversity Studies, University of Łódz, Banacha 1/3, PL-90-237 Łódź, Poland; tomasz.janiszewski@biol.uni.lodz.pl; 5 Department of Preclinical Sciences and Infectious Diseases, Poznań University of Life Sciences, Wołyńska 35, PL-60-637 Poznań, Poland; szeptycki.jan@gmail.com; 6 Weterynaryjne Laboratorium Diagnostyczne, Lab-Vet sp z o.o., Okrężna 8, PL-62-080 Tarnowo Podgórne, Poland; wilczynski@lab-vet.com.pl

**Keywords:** epidemiology, *Salmonella*, zoonosis, One Health

## Abstract

**Simple Summary:**

Bird feeders are known to be a transfer site for many important bird pathogens, such as zoonotic *Salmonella* spp., known to be widespread among wild birds in Poland. The aim of the study was to investigate (1) whether feeders can be a source of *Salmonella* spp., (2) whether the risk is the same for feeders located in cities and rural areas and (3) whether there is a different level of contamination with *Salmonella* spp. between old and new feeders. Data were collected in winter 2018 in Poland, and in total, 204 feeders were sampled. Material for analysis consisted of the remains of food and feces. We did not find the presence of Salmonella spp. in any of the tested samples collected from bird feeders. Reasons for the isolation of *Salmonella* spp. from feeders not being successful lie in the low intensity of bacterial shedding by infected wild birds and low survival of bacteria in the environment.

**Abstract:**

Bird feeders are known to be a transfer site for many important bird pathogens, such as zoonotic *Salmonella* spp., known to be widespread among wild birds in Poland. The aim of the study was to investigate (1) whether feeders can be a source of *Salmonella* spp., (2) whether the risk is the same for feeders located in cities and rural areas and (3) whether there is a different level of contamination with *Salmonella* spp. between old and new feeders. Data were collected in the period 12 January–28 February 2018 in four cities in Poland and nearby rural areas. In total, 204 feeders were sampled. The samples from feeders were taken after a 2-week period of feeding birds. Material for analysis consisted of the remains of food and feces. We did not find the presence of *Salmonella* spp. in any of the tested samples collected from bird feeders. Therefore, the estimated value of the 95% confidence interval for the binary data was 0.000–0.018. Reasons for the isolation of *Salmonella* spp. from feeders not being successful lie in the low intensity of bacterial shedding by infected wild birds and low survival of bacteria in the environment in bird feces—which are still not well studied.

## 1. Introduction

Along with the progressive urbanization, the observation of plants and animals in the city surroundings is the only opportunity for many people to interact daily with nature [1]. One of the activities allowing this is bird feeding, which has been well investigated to have a positive effect on the health, well-being and ecological awareness of people [2]. Despite the fact that bird feeding is a popular practice in many countries, numerous aspects of its impact on bird and human populations have not yet been well investigated [3]. Due to gaps in knowledge, bird feeding has contradictory reputations. Some see feeding birds as a pro-environmental activity, helpful for the survival of many species [4], while others emphasize the epidemiological risks associated with feeding and the typically low nutritional value of bird food [5].

Feeding birds entails some threats [5], the most important being the increased exposure of birds to pathogens and the higher risk of pathogen transmission between them. This is favored by the high density of bird gatherings in feeding places, the contact of birds of many different species in feeding places in combinations that are unlikely to occur in nature, and usually the poor hygiene of feeders and other feeding places, leading to significant microbiological contamination [6,7]. In addition, it has been studied that the density and competition appearing between birds in feeders can lead to severe stress and, as a result, immunosuppression, which in turn contributes to greater sensitivity to pathogens [7,8].

Feeders have been found to be an important transfer site for many avian pathogens, such as *Salmonella* spp., *Campylobacter* spp., *Mycoplasma gallisepticum*, *Macrorhabdus ornithogaster*, *Trichomonas* and Poxvirus avian virus [4,5,6,8,9,10,11]. Many pathogens transmitted by birds have a high zoonotic potential and, therefore, close contact between birds and their pathogens at feeding sites can pose a serious public health risk [12]. Among them, one of the most important is bacteria from the genus *Salmonella*. They are commonly found in wild birds, very often with no signs of infection and can be shed in feces [13]. Many outbreaks of salmonellosis in bird populations have been linked to using bird feeders [12]. It has been suggested that especially old feeders that have been used for a long period of time and kept in bad hygiene conditions can act as a source of infection of *Salmonella* spp. in birds [10,14]. Some studies have linked outbreaks of salmonellosis, also caused by highly pathogenic serovar *Salmonella* Typhimurium (*S*. ser. Typhimurium), in wild birds with outbreaks of salmonellosis in human populations [15,16]. In 1993–2012, similar temporal and spatial trends in *S.* ser. Typhimurium infection in Great Britain were found in the population of garden birds and humans. These data support the hypothesis that garden birds are the main reservoir of *Salmonella* infection. Most of the identified outbreaks of passerine salmonellosis occurred in and around the feeding sites of the birds. It has been suggested that bird feeding sites, especially dead birds and their droppings, may play a significant role in the transmission of salmonellosis to humans and increase the risk of infection [5,15]. It is believed that birds living in cities can act as disseminators of *Salmonella* spp., partly because pollution, microclimate and specific food in urban ecosystems affect bird immunity [17]. Birds living in rural areas, however, may differ greatly in terms of infection prevalence and dissemination, mainly due to differences in the structure of urban and rural habitats, human activity, species composition of birds and presence of farm animals [18,19]. Diversification in the degree of transmission of salmonellosis in urban areas may result from the greater concentration of birds in winter in urbanized areas, especially in unfavorable weather and food conditions [18,20]. Moreover, bird feeders in urban areas are a place of meeting for birds from a wide variety of species, coming from large areas and different populations [20].

In this regard, the aims of our study were to investigate whether bird feeders can act as a source of infection with bacteria *Salmonella* spp.; whether there is a difference between *Salmonella* spp. presence in feeders located in cities and rural areas; and whether there is a different level of contamination with *Salmonella* spp. between already used, old feeders (used for approximately 3–10 years) and new ones, provided specifically for the needs of the study. Salmonellosis has been reported as an important cause of mortality of garden birds, as *Salmonella* ssp. is transmitted to birds from direct contact with other infected individuals or through exposure to contaminated surfaces. During wintertime, birds of various species, especially granivorous and gregarious passerine species concentrate in feeding places [9,10], such as bird feeders in both urban and rural areas.

## 2. Materials and Methods

### 2.1. Field Study

Data were collected twice during winter, in the period 12 January–28 February 2018, in 4 cities in Poland (Bydgoszcz, Poznań, Olsztyn, Łódź). Urban sites were matched (up to a distance of 20 km) with a nearby rural area (according to earlier design presented in [18]). The particular period chosen for studies can be classified as a typical winter period in Poland with an average temperature of −3 °C, and snow cover up to 6 cm (www.imgw.pl; accessed on 17 June 2021); however, during December and January, temperatures were higher (with average temperatures of 4 °C, and generally no snow cover).

**Figure 1:**
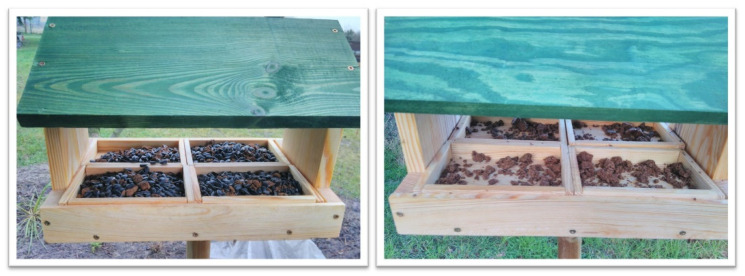
Experimental bird feeders used for investigating *Salmonella* spp. prevalence in winter in Poland. Please note the situation before (**left**) and after (**right**) providing sunflower seeds.

In total, we sampled 204 bird feeders (Bydgoszcz: 64; Poznań: 48; Olsztyn: 52; Łódź: 40), half located within urban areas and half located within rural areas. Some of the sampled feeders were old (Bydgoszcz: 32; Poznań: 24), which were provided 3–10 years ago and regularly maintained by people. Other feeders (n = 148) were new and experimental, contained at the bottom four trays with sunflower seeds, very popular during winter bird feeding in Poland [21]. Each experimental feeder had the same design—the shape of a small house with a roof (Figure 1). Samples of bird feces for microbiological analyses were taken after two weeks of regularly providing food to the feeders.

The samples from feeders consisted of the remains of food and feces. Fecal samples and food remnants were collected from trays on which food was placed, and feces were also collected from the roofs of the feeders. Loose material was poured into sterile containers. Additional material was scraped from the roof of the feeders with sterile feces swabs into sterile containers. Containers were marked with an individual code (unknown for the laboratory) and sent for testing.

### 2.2. Laboratory Analysis

Isolation of Salmonella was carried out according to the PN-EN ISO 6579: 2003 + AC: 2014-11. Media used for incubations included:

1. Nonselective pre-enrichment medium—Buffered Peptone Water (Biomerieux—**** Marcy l’Etoile, France);

2. Selective enrichment medium—Modified Semisolid Rappaport Vassiliadis medium—MSRV (Graso—Starogard Gdański, Poland);

3. Solid selective media—Salmonella Chrom Agar SAL-P (Graso), Briliant Green Agar—BGA (Graso— Starogard Gdański, Poland).

Portion of collected material was added to plastic bags and then diluted in a ratio of 1:10 with Buffered Peptone Water. The inoculated bags were homogenized and incubated for 18+/−2 h at 36 °C+/−2 °C. After incubation, samples were cultured on a plate with MSRV medium by adding 100 µL bacterial culture in three drops. The plates were incubated at 41.5+/−1 °C for 48+/−2 h. Typical growth on the plates (grey-white turbid zone extending out from the inoculated drop) were cultured onto agar medium BGA and Chrom agar. Then, plates were incubated at 37+/−1 °C for 24+/−2 h.

### 2.3. Statistical Analysis

After obtaining all laboratory results, trays for binary data (presence–absence of *Salmonella* spp.) were calculated using the binomial (Clopper-Pearson) “exact” method [22].

## 3. Results

We did not find the presence of *Salmonella* spp. in any of the 204 samples collected from bird feeders. Therefore, the estimated value of the 95% confidence interval for the binary data is 0.000–0.018.

The absence of Salmonella in all samples obviously prevents comparisons between trials (rural vs. urban places and new vs. old feeders). Therefore, the probability of infection by Salmonella is very low.

A wide variety of birds regularly visited the bird feeders, comprising the following 16 species: Chloris chloris, Coccothraustes coccothraustes. Columba livia f. urbana, Corvus monedula, Cyanistes caeruleus, Dendrocopus major, Garrulus glandarius, Parus major, Passer domesticus, Passer montanus, Periparus ater, Pica pica, Poecile palustris, Sitta europea, Streptopelia decaocto and Turdus merula.

## 4. Discussion

Bacteria of the genus *Salmonella* are among the most important zoonotic pathogens. They are widespread in the environment and characterized by high genetic variability. Many species and serovars of *Salmonella* are found in the digestive system of wild animals, including birds [23]. Salmonellosis in birds can occur due to temporary colonization of the digestive tract by strains from the environment or can be caused by species-specific strains, which may or may not be pathogenic [13]. Therefore, birds infected with *Salmonella* spp. can developed signs of disease, die or remain asymptomatic and shed bacteria, especially under stress conditions, such as cold weather or competition with other birds in feeding sites [8,24]. Numerous studies indicate that wild birds may be a reservoir and disseminator of *Salmonella* spp. [25,26].

Salmonellosis is often a cause of mortality in wild bird populations [27]. Epizooties of *S*. ser. Typhimurium in wild birds were observed in Switzerland in the 1950s [28] and in eastern North America from winter 1997 to summer 1998 [29]. An outbreak caused by *S*. ser. Typhimurium was also noted in the Norway in the years 1999–2000, when infection with this pathogen was detected in 64.8% of wild Passeriformes found dead in bird feeding stations located across the country [30]. It is also known that wild birds can be a source of human infections with *Salmonella* spp. An outbreak of S. ser. Typhimurium in humans and cats, associated with infections and mortality in wild birds, was reported in 1999 in the center of Sweden [31]. Similarly, the same strains of *S*. ser. Typhimurium were isolated from wild bird populations and people in Germany [32]. Large-scale 20-year studies conducted in England and Wales showed that strains of the *S*. ser. Typhimurium found in humans and in wild garden birds were very closely related [15]. Moreover, most of the recorded outbreaks of avian salmonellosis were found in or around the areas of feeders. Research from New Zealand also linked the outbreak of salmonellosis caused by *S*. ser. Typhimurium in the human population with the outbreak of salmonellosis in wild birds [16].

For these reasons, it is important to better understand in which places wild birds can become infected with *Salmonella* spp. and in which ways avian bacteria can come into contact with people. One of the potentially perfect and often suggested places to investigate this is bird feeders [5,19]. It is worth underlining that all of the bird feeders used in our study were not regularly cleaned. Feeders represented the level of hygiene commonly found in bird feeders across the country, which is usually poor [10]. It is then surprising that despite the fact that *Salmonella* spp. are known to be widespread in wild bird populations in Poland [10,26], no evidence of the presence of this pathogen in a large number of bird feeders in our study was found. Similar results were obtained in a study conducted in southwestern Ontario [33]. The epidemic of salmonellosis in some species of birds using feeders was observed there in the winter of 1997–1998. To investigate the source of infection, in the winter of 1999, samples of feed and feces for *Salmonella* spp. detection were taken from 124 bird feeders. Surprisingly, *Salmonella* spp. was not found in any of them. However, in a different study conducted in Scotland [34], *Salmonella* spp. bacteria were successfully isolated from bird feeders, also in winter months. This study covered two areas. In the first area, with a history of deaths in wild birds from salmonellosis in previous years, *S*. ser. Typhimurium was isolated from 33%, 42% and 48% of the pooled fecal samples collected from different parts of bird feeders. In the second area, without a history of salmonellosis in previous years, *S*. ser. Typhimurium was found in only 2% of feces from one specific part of feeders. Therefore, *Salmonella* spp. shedding can differ greatly in bird populations living in different areas. It is also interesting that feces from different parts of bird feeders had different bacterial burdens. This could indicate that even small differences in conditions in areas of the feeder can influence the survival of bacteria.

There is a lack of detailed information about the duration and intensity of bacterial shedding by infected wild birds and the survival of *Salmonella* spp. in the environment in bird feces [35]. This may be why *Salmonella* spp. may be hard to find in some conditions at feeding sites. There is very little information about the intensity and time of shedding of naturally occurring bacteria in wild birds. In one study, wild bird feces (of herring gull) have been found to have concentrations of *Salmonella* spp. in a range from 22 MPN/g to 2.4 × 109 CFU/g [35]. Another study, also regarding herring gulls, showed that the time of bacteria shedding was very short—up to 4 days [36]. It worth underlining that there can be wide variation in *Salmonella* spp. shedding intensity and time among different avian taxa. Due to these, we still do not know the exact mechanisms of shedding bacteria by wild birds of species usually visiting feeders. There are also very limited data about the survival of *Salmonella* spp. in wild bird feces. Some information has been obtained from studies on Canada geese. A 28-day trial conducted in parks in London, England, showed that bacteria from inoculated Canada goose droppings with 10^4^–10^5^ CFU/g of *Salmonella* spp. survived the 28-day trial, in conditions of heavy rain [37]. It is, however, possible that the survival of *Salmonella* spp. in the feces of Canada geese is greater than the survival in feces of smaller bird species, observed in bird feeders. Their feces are smaller, with a greater relative surface area, and as a consequence, bacteria shed in them are more prone to desiccation. This hypothesis, however, has not yet been tested experimentally [35].

Another reason that the isolation of *Salmonella* spp. from bird feeders was not successful in our study could be the temperature in the study period. As commonly reported, the lowest temperature allowing *Salmonella* spp. survival is 4 °C [38]. However, some data show that *Salmonella* spp. can survive at an even lower temperature [39]. Temperatures below 4 °C strongly inhibit the metabolism of bacteria, making cells inactive. In this state, Salmonella spp. cell can survive even at freezing temperature [40]. The isolation method we used in our study, preceded by peptone propagation, allows the activation of cells and subsequent culture in media. In this regard, the lack of isolated *Salmonella* spp. was probably not caused by the temperature in the study period we chose. Bird feeders are not commonly used in Poland during warmer times of the year (spring and summer). However, we do not exclude the possibility that conducting a *Salmonella* spp. survey in these seasons could produce different results.

## 5. Conclusions

A recent review and metanalysis highlighted that the current available literature is not sufficient to estimate the likelihood of enteric pathogen spillover from wild birds to humans, and the risk of such an event may be overestimated [35]. Our study may lead to a similar conclusion. We need more data to fully understand how zoonotic pathogens can be spread and transmitted among wild bird and human populations.
